# Supplementary material for: An Ultra-Short Measure of Excessive Daytime Sleepiness Is Related to Circadian Biological Rhythms: The French Psychometric Validation of the Barcelona Sleepiness Index
Source: J Clin Med. 2022 Jul 4;11(13):3892. doi: 10.3390/jcm11133892 (PMC9267909; doi:10.3390/jcm11133892)
Supplement: Supplementary file 1 [file jcm-11-03892-s001.zip › jcm-1753987-supplementary.pdf]

Supplementary Table S1. Subgroup correlation analyses

	**All (n = 662)**	**Women (n = 503)**	**Men (n = 159)**	**Freshmen (n = 274)**	**Others (n = 388)**
ESS score	0.47*	0.49*	0.41*	0.44*	0.49*
THAT score	−0.40*	−0.42*	−0.33*	−0.42*	−0.39*
ISI score	0.36*	0.41*	0.17†	0.37*	0.35*
Anxiety symptoms	0.15*	0.20*	−0.04	0.17#	0.14#
Depressive symptoms	0.24*	0.25*	0.22#	0.24*	0.24*
Mean sleep duration	−0.11#	−0.13#	−0.07	−0.13†	−0.10†
Mean sleep efficiency	−0.10#	−0.17*	0.08	−0.16#	−0.07
Social jetlag	0.09†	0.12#	0.01	0.11	0.08

†$p < 0.05$
#$p < 0.01$
*$p < 0.001$

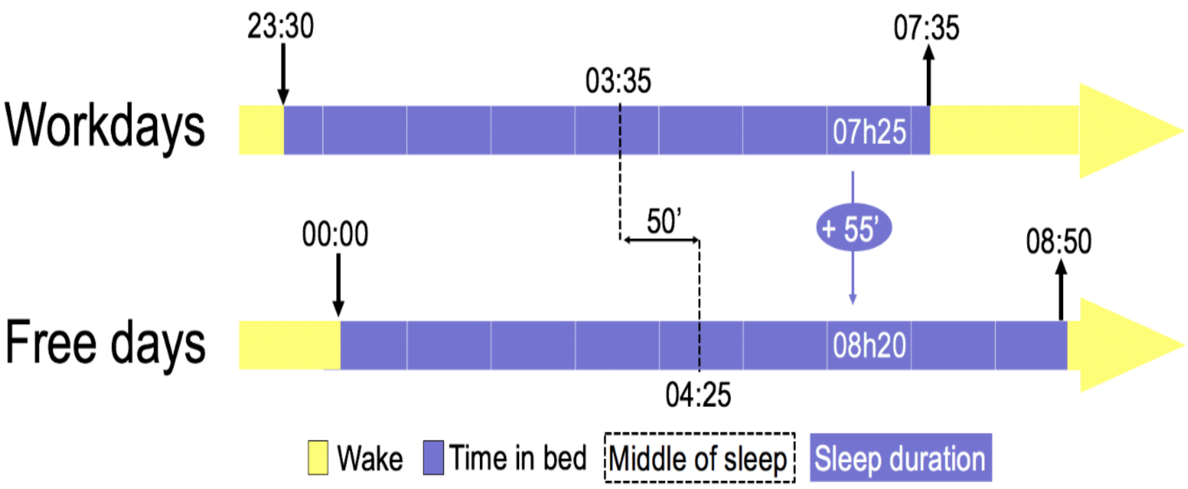

Supplementary Figure S1. Sleep schedules (n = 662).

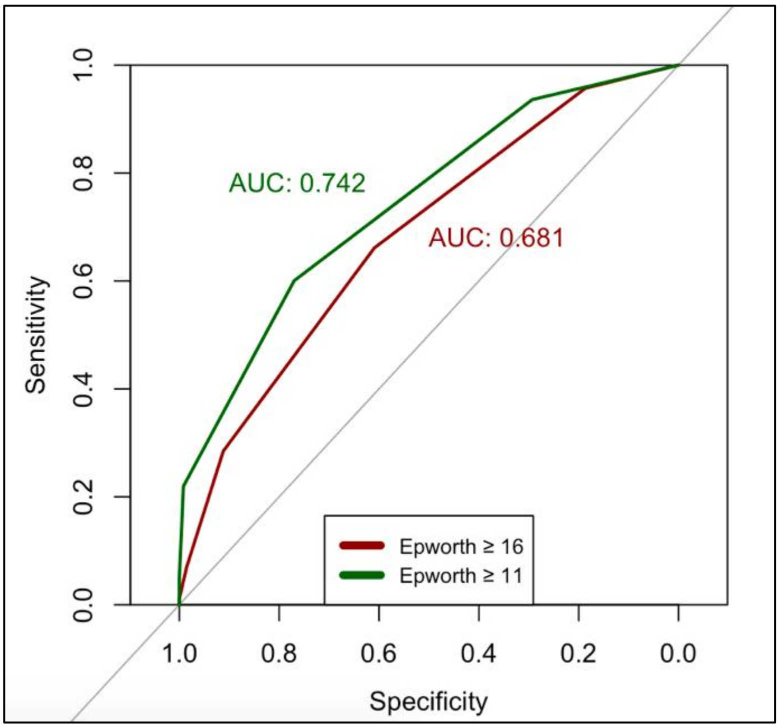

Supplementary Figure S2. Receiver operating characteristic analysis of the ability of BSI score to predict EDS, according to ESS score.
